# Plating and Stripping Calcium Metal in Potassium Hexafluorophosphate Electrolyte toward a Stable Hybrid Solid Electrolyte Interphase

**DOI:** 10.1021/acsaem.3c00098

**Published:** 2023-03-30

**Authors:** Paul Alexis Chando, Jacob Matthew Shellhamer, Elizabeth Wall, Wenlin He, Ian Dean Hosein

**Affiliations:** Department of Biomedical and Chemical Engineering, Syracuse University, Syracuse, New York 13244, United States

**Keywords:** calcium, batteries, anode, electrolyte, solid−electrolyte interface

## Abstract

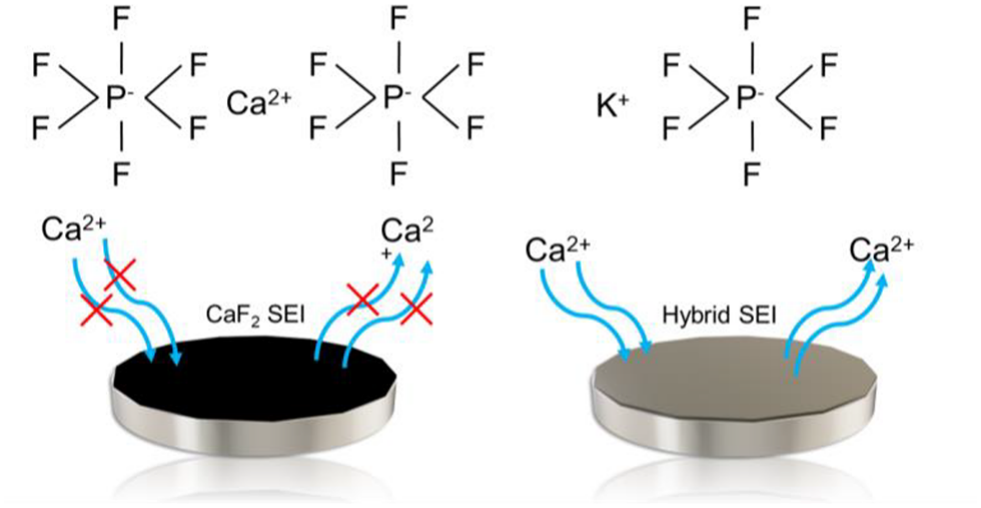

The use of calcium (Ca) metal anodes in batteries is currently challenged by the development of a suitable solid electrolyte interface (SEI) that enables effective Ca^2+^ ion transport. Native calcium electrolytes produce a passivation layer on the surface of the calcium electrodes during cycling, causing a decrease in capacity during cycling and the need for large overpotentials. The use of a hybrid SEI is a strategy to mitigate the uncontrolled production of a passivation layer and reduce the overpotentials needed for the plating and stripping of calcium. Here, we report the development of a hybrid potassium (K)/Ca SEI layer investigated in symmetric Ca//Ca cell configurations. Using KPF_6_ salt in a ternary mixture of carbonate solvent (EC/EMC/DMC), Ca//Ca cells can be cycled up to 200 h at a capacity of 0.15 mAh/cm^2^ with a current density of 0.025 mA/cm^2^. The symmetrical cells consistently cycle at overpotentials of 1.8 V. Ex-situ X-ray diffraction (XRD) of cycled electrodes reveals plating and stripping of both calcium and potassium. Energy dispersive X-ray (EDX) maps confirm the plating of calcium and potassium during galvanostatic cycling. Scanning electron microscopy (SEM) cross-sectional views of the calcium electrodes reveal a continuous SEI layer formed over the calcium metal. XRD analysis reveals the SEI layer consists of K-based inorganics along with the identification of permanent and transient phases. FTIR outlines the parallel plating of both calcium and potassium at both regions of redox activity. Raman spectroscopy of the electrolyte reveals compositional changes over the course of cycling that promote increased plating and stripping. The results indicate that potassium electrolytes are a possible route for tuning the SEI to enable reversible calcium electrochemical cycling.

## Introduction

The ever-increasing energy demand from developing technologies, along with the limited supply of lithium to meet them, has necessitated the need to explore post-lithium-ion battery solutions.^1^ Divalent ions such as calcium are particularly attractive due to their similar reduction potentials to lithium and wide availability, making the economics associated with its use feasible. While there are such benefits to the use of calcium, there are challenges that need to be addressed such as the identification of appropriate electrolytes to use with calcium metal. Electrolytes for calcium metal batteries have been limited by successful plating and stripping. The cause of such bottlenecks has been the passivation layer that forms on the calcium metal surface as plating continues with native calcium electrolytes (i.e., employing only calcium salts). The SEI is formed from the degradation of the electrolyte onto the surface of the calcium, functioning as an electrically insulating material while still being ionically conductive. The use of fluorinated electrolytes produces a continuous deposition of calcium fluoride (CaF_2_) onto the SEI of the calcium which acts as highly insulating materials, decreasing ionic conductivity of the SEI and ultimately reducing the capacity on subsequent cycles.^2^ This issue was first addressed by running plating and stripping of calcium at elevated temperatures.^3^ The use of elevated temperatures increased the ionic conductivity of the SEI and slowed the development of the passivation layer. The successful plating and stripping of calcium from such efforts renewed interest in exploring alternative solutions with electrolyte formulations.^4−8^

An alternate strategy that bypasses the need for elevated temperatures and high overpotentials is the use of a hybrid SEI. The use of mixed cations in the SEI has proven effective at cycling with lithium anodes.^9^ Expanding the use of the hybrid SEIs to calcium has focused on using cations with similar atomic radii such as sodium (Na) and continuing the use of hexafluorophosphate anions due to their weakly coordinating nature.^10^ Sodium hexafluorophosphate (NaPF_6_) was used as the salt in an electrolyte with calcium metal to create a sodium oxide (Na_2_O) phase in the SEI that allowed the deposition of calcium while mitigating the continuous formation of CaF_2_ that would occur with a native calcium based electrolyte.^4^ The efficacy of such an approach with sodium has prompted additional efforts at tailoring the SEI for improved cycling efficiencies.^11,12^ An additional possibility for hybrid SEIs with calcium metal is the use of potassium owing to its larger ionic radius to calcium. Currently, potassium is a largely unexplored option for hybrid SEIs with calcium. The existing work on it has focused on having potassium partake in composite SEI along with sodium and calcium.^13^ While successful, an evaluation of the standalone hybrid SEI between potassium and calcium remains unexplored.

Here, we report the development of such a hybrid SEI using potassium hexafluorophosphate (KPF_6_) salt in a composite solvent of ethylene carbonate (EC), dimethyl carbonate (DMC), and ethyl methyl carbonate (EMC) as it is cycled between calcium metal electrodes. The plating and stripping behavior observed maintained overpotentials below 2 V throughout the course of cycling, while also exhibiting discrete events of potassium and calcium deposition onto the electrodes. XRD characterization of the SEI that formed on the electrodes revealed a composite of phases that allow the plating and stripping of calcium. Phase composition of plated materials was further supported by FTIR and SEM/EDX analysis on the deposited materials.

## Experimental Section

### Materials

Potassium hexafluorophosphate (KPF_6_, ≥99%), calcium (Ca, 99%) granules, and molecular sieves (3 Å, 4–8 mesh) were purchased from Sigma-Aldrich. Calcium granules were first flattened to an 8 mm diameter and 0.5 mm thickness with a mechanical press. The calcium pieces were subsequently polished with a dremel (4300, Dremel). Gold electrodes (99.95%) were purchased from Goodfellow Cambridge Limited. Additionally, ethylene carbonate (anhydrous, 99%), dimethyl carbonate (anhydrous, ≥99%), and ethyl methyl carbonate (99%) were purchased from Sigma-Aldrich.

### Electrolyte Preparation

All electrochemistry experiments used a 1 M solution of KPF_6_ in EC/DMC/EMC. The solvents were first mixed in a 1:1:1 volume ratio for 24 h before being dried for 48 h over molecular sieves. The KPF_6_ was dried overnight in a vacuum oven at 120 °C before being added to the solvent mixture. The KPF_6_ solution was stirred for 24 h to allow full dissolution of the salt and was again dried over molecular sieves. Water content of the electrolyte was verified by a Karl Fisher Titrator (899 Coulometer, Metrohm) to be below 50 ppm. All experiments were performed in a glovebox where H_2_O and O_2_ levels were below 0.5 ppm.

### Electrode Preparation

The calcium pellets were first flattened to a diameter of 8 mm and thickness of 1 mm using a mechanical press. The calcium electrodes were then polished with a Dremel 4300 using a procedure found elsewhere.^8^ All polishing procedures were performed in a glovebox with O_2_ and H_2_O levels below 0.5 ppm. Briefly, a wire brush was first used to remove the oxide layer on the calcium disc. Once the initial oxide layer was removed, a dremel (Dremel 4300) with a silicon carbide (SiC) tip was used to polish the calcium to achieve a mirror finish. The dremel was operated between 5K and 15K rpm to achieve a highly polished finish on the Ca electrodes. For electrochemical experiments using a gold (Au) electrode, the electrode was electrochemically cleaned in a sulfuric acid bath before being rinsed with methanol and dried under ambient conditions.^14^

### Electrochemistry

A beaker cell was used in all experiments. Calcium electrodes were attached to the end of 316 stainless steel supports and immersed in the electrolyte. Two-electrode configurations were used for plating and stripping. Calcium or gold was used as the working electrodes (WE) and calcium as the counter (CE) and reference (RE) electrodes. Following the immersion of the electrodes into the electrolyte, the plating and stripping experiments were performed with a Metrohm Autolab at a current density of 0.025 mA/cm^2^ and an areal capacity of 0.15 mAh/cm^2^. Linear stability window measurements were made using a two-electrode cell with gold as the blocking electrode and calcium as the nonblocking electrode. Measurements were made at a sweep rate of 0.5 mV/s with a voltage window from 0 to 5 V. Cyclic voltammetry was performed after 10 plating and stripping cycles (0.025 mA/cm^2^, 0.15 mAh/cm^2^) with a calcium symmetric cell configuration. The 10 cycles of plating and stripping were used to form the hybrid SEI on the calcium electrodes. The scan rate used was 0.5 mV/s with a voltage range from −3 to 3 V. The evolution of the hybrid SEI on calcium electrodes was recorded using in situ electrochemical impedance spectroscopy (EIS). The measurements were performed with a Solartron Energy Lab XM Instrument over a frequency range from 0.1 Hz to 1 MHz between each plating and stripping step. A 10 mV perturbation was used in all EIS measurements, and the resulting Nyquist plot was analyzed using an equivalent circuit model.^5,6,15,16^ Transference number measurements were performed using the Bruce and Vincent method.^17,18^ A 10 mV potential was applied to a calcium symmetric cell, and the current response over time was recorded. The contact resistance of the calcium electrodes was recorded with impedance measurements and determined using an equivalent circuit model. The transference number of the potassium hexafluorophosphate electrolyte was calculated with the following formula:
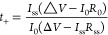
where *I*_ss_ is the steady-state current, *I*_0_ is the initial current, Δ*V* is the applied potential, *R*_ss_ is the steady-state resistance, and *R*_0_ is the initial resistance.^17−20^

### Electrode Characterization

A Rigaku Miniflex diffractometer was used for the collection of X-ray diffraction data. All samples analyzed under XRD were washed with solvent following experiments. Cu Kα radiation was used to collect diffraction data in the range of 10°–80° at a scan rate of 5°/min. All XRD measurements were performed at room temperature. XRD analysis of each material was performed using Highscore Plus software.^21^ The Materials Project was used as well for identification of phases.^22^ Scanning electron microscopy (SEM) was performed with a JEOL 5600 and was equipped with an energy dispersive X-ray (EDX) detector. The accelerating voltage used on the samples was 15 kV. All samples analyzed with the SEM were sputter-coated with gold. FTIR analysis of the samples was performed in transmission mode at 4 cm^–1^ resolution with an ATIR-FTIR spectrometer (Bruker, Alpha). Raman spectroscopy was used for evaluating the compositional changes of the electrolyte during plating and stripping between the calcium symmetric cell experiments. All measurements were performed with a confocal microscope connected to a Raman spectrometer (Renishaw InVia).

## Results and Discussion

**Figure 1:**
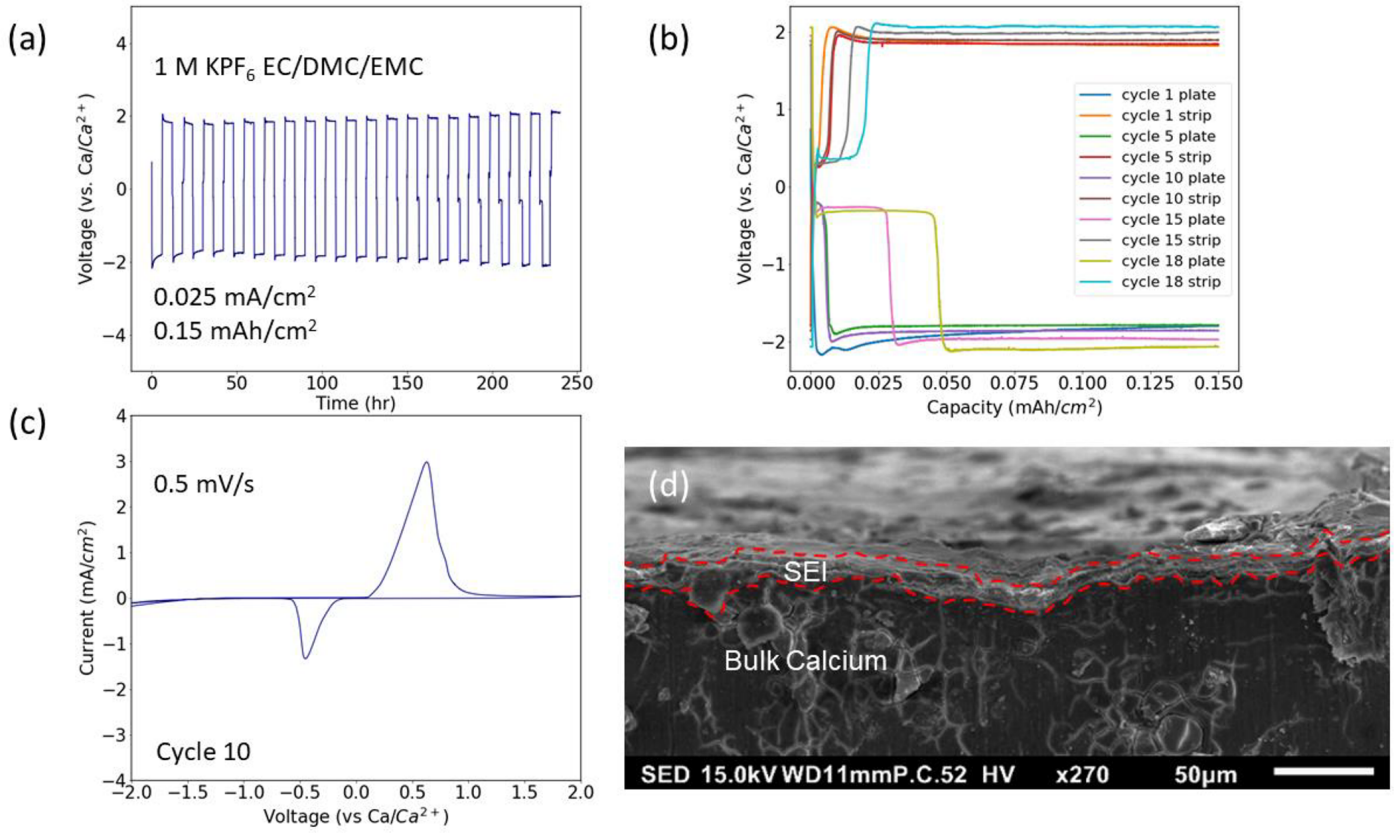
Galvanostatic cycling. (a) Plating and stripping of the Ca//Ca symmetric cell. (b) Voltage vs capacity curves. (c) CV of the Ca//Ca symmetric cell. (d) Cross-sectional of SEI on calcium electrode after 10 cycles of galvanostatic cycling.

Figure 1a details the plating and stripping behavior of the calcium symmetrical cell in 1 M KPF_6_ EC/DMC/EMC electrolyte. The overpotentials from plating and stripping remain at 1.8 V (vs Ca/Ca^2+^) for over 20 cycles. Unlike plating and stripping experiments that use Ca(PF_6_)_2_ where the overpotentials quickly rise to 5 V,^4^ cycling with KPF_6_ is far more stable. Plateaus at −0.2 and −1.8 V can be observed in the experiments, consistent with behavior that has been seen previously with hybrid SEI plating and stripping.^13^ The galvanostatic cycling for the symmetric cell was set to an areal capacity of 0.15 mAh/cm^2^_._ The capacities are summarized in Figure 1b with the −0.2 V potential plating with an areal capacity of 0.0085 mAh/cm^2^ and the −1.8 V potential plating with an areal capacity of 0.1415 mAh/cm^2^. The capacities from the plating and stripping at 0.2 V overpotentials increased over the course of cycling and ultimately reached a capacity of 0.05 mAh/cm^2^ at 0.3 V overpotentials. Additional plating and stripping studies using a separate reference electrode were found to decrease overpotentials from 1.8 to 1.7 V (vs Ca/Ca^2+^). Measurements taken of the symmetric cell working electrode during stripping were recorded to be 1.6 V (vs Ca/Ca^2+^) while the counter electrode was measured with a potential of −0.07 V (vs Ca/Ca^2+^). Additionally, the working electrode potential is −0.07 V (vs Ca/Ca^2+^), and the counter electrode potential is 1.6 V (vs Ca/Ca^2+^) during plating. The results from the three-electrode are summarized in Figure S1. Cyclic voltammetry studies were performed to correlate faradaic reactions with the observed plating and stripping behavior. The results from the cyclic voltammetry study are outlined in Figure 1c. The voltage scans identify a redox-active process on the calcium electrodes with an onset potential of −0.2 V and a maximum current response at −0.5 V. A cross section of the SEI formed on the calcium metal electrodes was analyzed under SEM imaging and was found to have formed an SEI thickness of 9 μm (Figure 1d).

**Figure 2:**
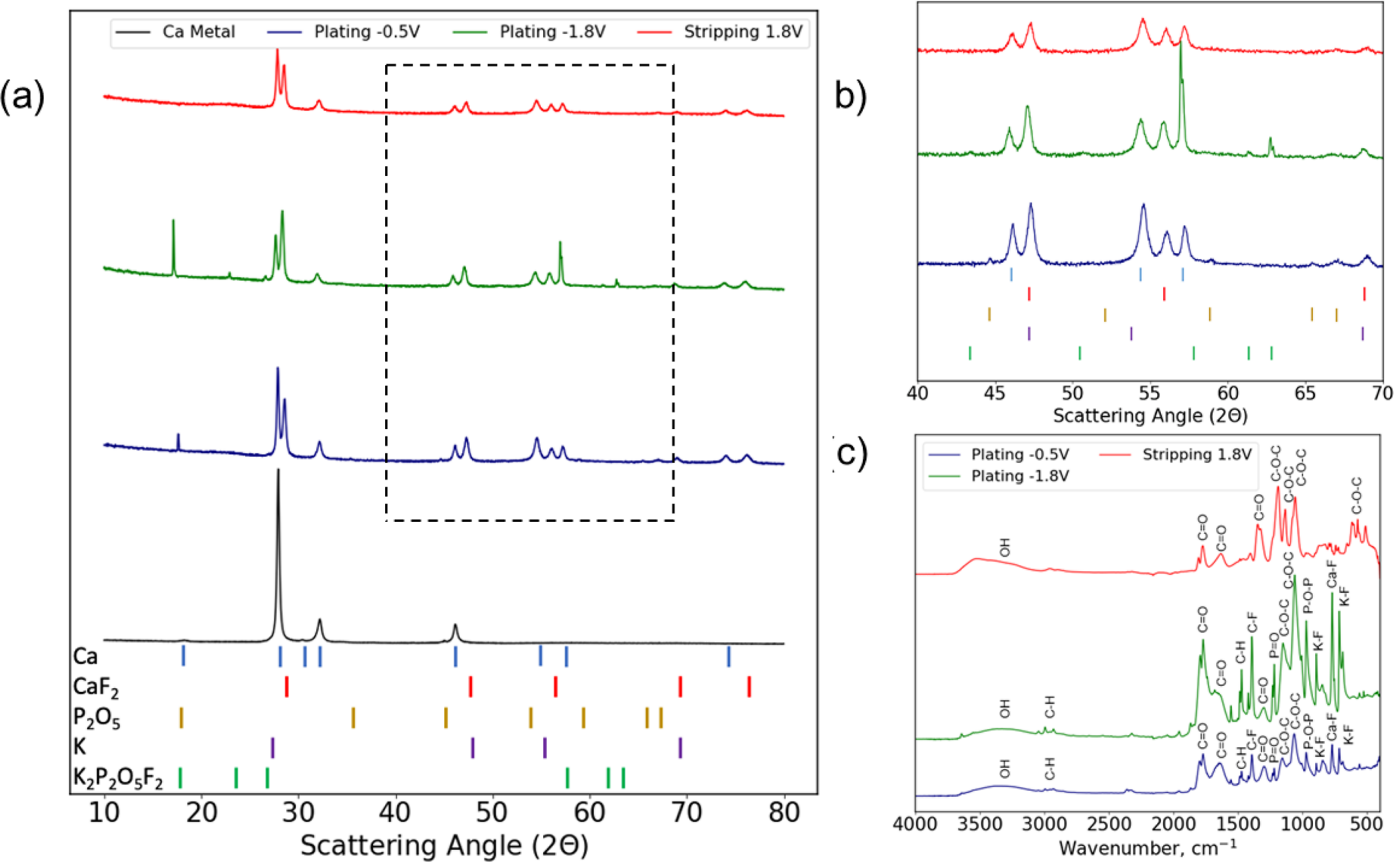
XRD of galvanostatic cycling with calcium electrodes. (a) XRD profiles of plating at −0.5 and −1.8 V along with XRD of stripping at 1.8 V in a calcium symmetric cell. (b) XRD profiles of plating and stripping between the diffraction angles of 40°–70°. (c) FTIR on the calcium plated and stripped materials.

To characterize the plating and stripping behavior observed in Figure 1a, ex-situ XRD studies were performed on the calcium electrodes. Potentiostatic holds at the previously observed voltages were performed to run controlled plating and stripping experiments on the calcium electrodes. Once completed, the electrodes were placed inside the Rigaku MiniFlex diffractometer and analyzed over the specified range of diffraction angles. The results from the XRD analysis are summarized in Figure 2a,b. Initial reflections of the calcium metal (mp-45) are recorded at 28°, 32°, and 46°. Additional reflections of calcium did not appear in the baseline scan due to the processing conditions of the electrode. Comparisons between processed and unprocessed calcium electrodes can be found in Figure S2. Following the plating potential at −0.5 V, new calcium (mp-45) reflections are observed at 54.5°, 57.2°, and 74°, revealing that the −0.5 V plating potential deposits calcium metal onto the SEI surface. The plating of calcium at −0.5 V is consistent with the cyclic voltammetry data and reveals that the redox reaction observed in Figure 1c was the plating and stripping of calcium. In addition to the new calcium reflections observed, phases of calcium fluoride (CaF_2_, mp-2741) and phosphorus pentoxide (P_2_O_5_, mp-562613) were detected on the calcium surface and are identified in Figure 2a,b. Phosphorus pentoxide reflections were observed at 17.5°, 35.5°, 44.5°, 52.9°, 58.8°, 65.5°, and 67°. The presence of the CaF_2_ phase in the plated material is an inevitable product of electrolyte decomposition onto the surface of the calcium metal electrode.^3^ The appearance of potassium (mp-58) as a plated material is observed after plating at the −1.8 V potential. Potassium plating reflections are identified at 26.7°, 47.1°, 54.4°, and 69.2°. While the reflections at 47.1°, 54.4°, and 69.2° overlap with calcium and calcium fluoride phases, the 26.7° reflection is unique to potassium and identifies it as a plated material onto the hybrid SEI surface. In addition to the previously defined phases, potassium difluorodiphosphate (K_2_P_2_O_5_F_2_, mp-558480) is also detected as a deposited material onto the hybrid SEI, and its reflections are observed at 17.1°, 22.9°, 26.7°, 57.1°, 61.4°, and 62.9°. The final component of the ex-situ XRD study was performing a stripping step to the calcium electrodes. After stripping the calcium electrodes, reflections associated with potassium and potassium difluorodiphosphate were removed. Reflections of calcium metal decreased after stripping. The reflections of P_2_O_5_ at 65.5° and 67°, while reduced in intensity, remained after stripping.

FTIR ex-situ studies were performed to complement the findings from XRD analysis. Focusing on the −0.5 V plating potential, the presence of calcium and calcium fluoride in the hybrid SEI surface is outlined from the Ca–F bond observed at 770 cm^–1^.^23^ Following the same behavior as the ex-situ XRD study, the P_2_O_5_ phase is also observed at the −0.5 V plating potential and is identified by the P–O–P bond at 970 cm^–1^.^24^ The P=O bond at 1210 cm^–1^ forms from the decomposition of the KPF_6_ electrolyte during the SEI formation.^25,26^ The degradation of the carbonate solvents in the electrolyte produce C–O–C bonds at 1067 and 1154 cm^–1^ along with C=O bonds at 1300, 1769, and 1800 cm^–1^.^25−28^ Additionally, carbonate degradation on the calcium electrode produces C–H bonds at 1475, 2900, and 3000 cm^–1^.^25,27^ Minimal water is observed through an O–H bond at the broad peak centered around 3350 cm^–1^.^25^ The plating potential at −1.8 V includes the previously determined bond assignments. The intensities of Ca–F, K–F, and C–F bonds all increase after plating at −1.8 V, indicating an increased deposition of materials consistent with the ex-situ XRD study performed on the calcium cycled in the symmetric cell. Plating of the potassium onto the calcium surface is identified with increased peak intensities at 717 and 892 cm^–1^.^29,30^ The increase of the Ca–F bond, based on its increase in intensity at −1.8 V, would have some overlap with the K–F bond as well. Lastly, the stripping at 1.8 V results in a decrease of the fluoride-based bond length intensities. The decrease of the fluorinated bonds, based on the ex-situ XRD, would coincide with the stripping of the K_2_P_2_O_5_F_2_. The primary peaks remaining on the calcium electrode poststripping are C–O–C bonds that were formed from the decomposition of the solvent onto the calcium surface along with small amounts of the P–O–P bonds from the P_2_O_5_ and Ca–F from CaF_2_. This is corroborated from the FTIR and XRD analyses that have been performed on the SEI.

**Figure 3:**
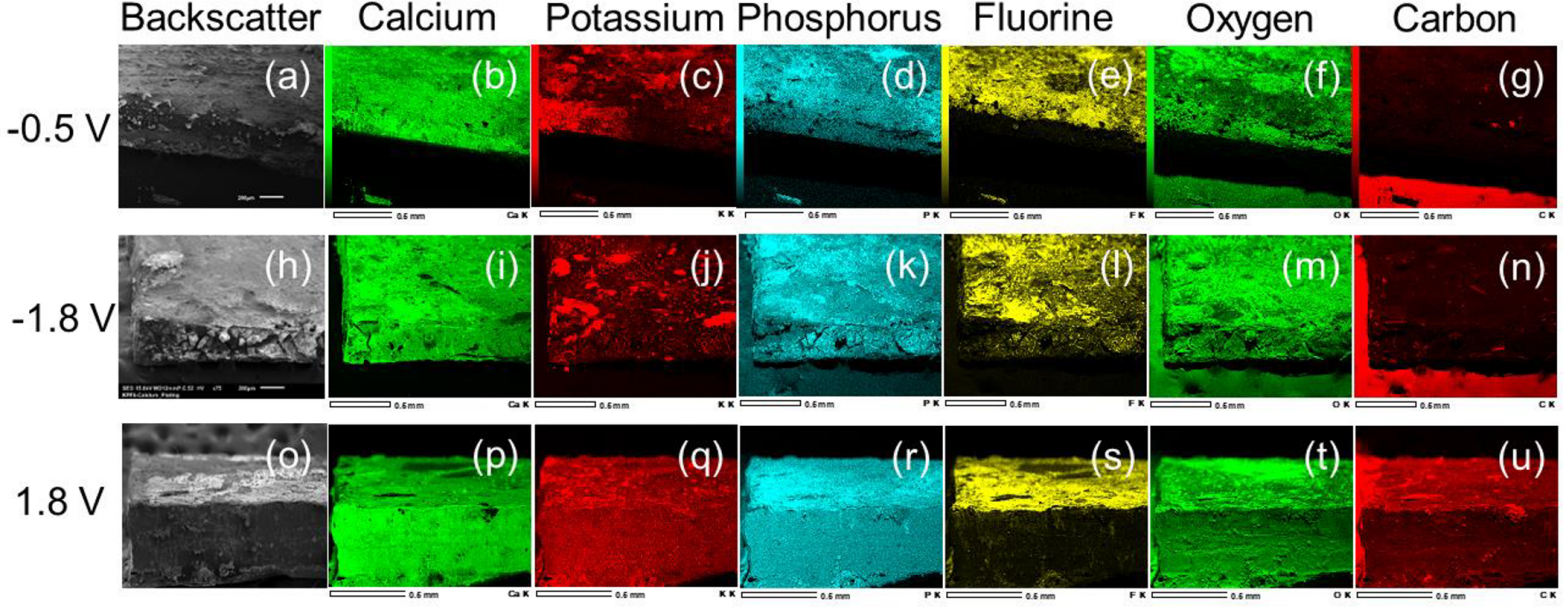
SEM/EDX from plating and stripping. (a–g) EDX of calcium electrode from −0.5 V plating. (h–n) EDX of calcium electrode from −1.8 V. (o–u) EDX analysis after stripping at 1.8 V.

The plating and stripping behavior was also analyzed by EDX mapping of a cycled Ca electrode. Figure 3 details the EDX map spectra observed for the two plating and stripping potentials examined. Figure 3a–g maps plating at −0.5 V. Focusing on the phases of the plated materials, the atomic signals for calcium, oxygen, phosphorus, and fluorine were 8.83%, 32.22%, 0.61%, and 13.45%, respectively. Beyond the plating at −0.5 V, the deposition of potassium onto the calcium electrode increases (Figure 3h–n). EDX of the potassium shows a strong increase in its fluorescence at this potential. While the atomic % signal of potassium remains below 1% at −1.8 V, this is influenced by the strong calcium signal (7.02%) from the bulk electrode and carbon (46.34%), minimizing compositional changes from the surface of the SEI. The phosphorus, oxygen, and fluorine signals from the −1.8 V plating were 0.52%, 36.52%, and 7.8%. The stripping from 1.8 V (Figure 3o–u) shows a decrease in fluorescence with the plated elements. The compositional changes in the calcium, oxygen, fluorine, phosphorus, and potassium signals were 17.05%, 44.8%, 11.34%, 0.09%, and 0.37%, respectively. From the analysis performed with EDX, all plated materials observed consist entirely of inorganic materials because no change in the carbon signal is observed throughout cycling (Figure 3g,n,u). To determine the thickness of deposition from each plating cycle, the K_2_P_2_O_5_F_2_ phase from the XRD was chosen because it is one of the phases fully stripped after applying the 1.8 V stripping potential. Focusing on the potassium EDX, it can be inferred, based on the atomic radius of potassium, that the deposition of each plating cycle is at least approximately 280–400 pm.

**Figure 4:**
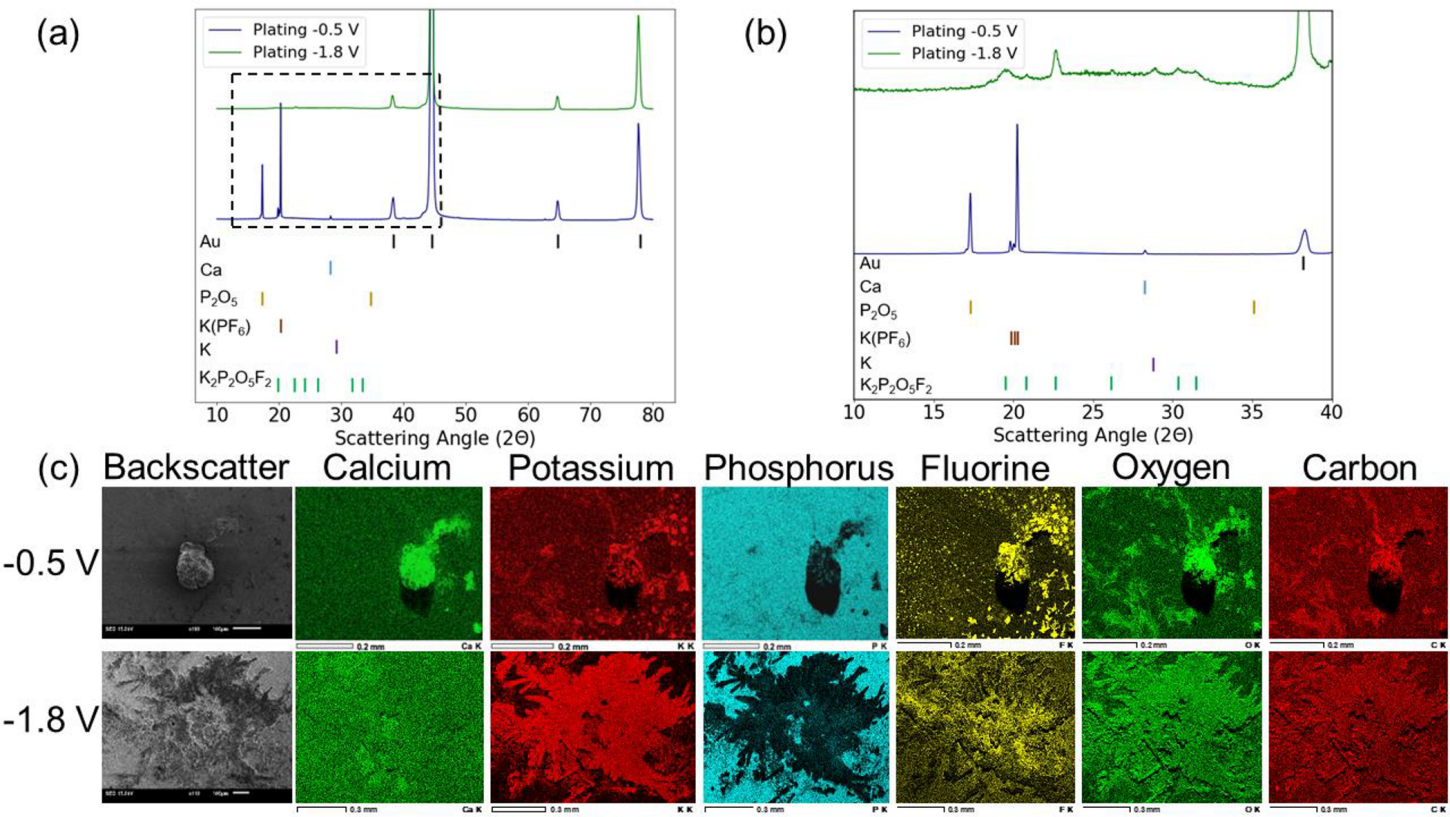
Plating onto gold electrode. (a) XRD profiles from plating onto a gold working electrode at −0.5 and −1.8 V. (b) XRD profiles from plating onto gold between diffraction angles of 10°–40°. (c) EDX of plated gold electrode at −0.5 and −1.8 V.

To further understand the plating behavior with the KPF_6_ EC/DMC/EMC electrolyte, a secondary cell was set up that used gold as a working electrode and calcium as a counter electrode (Au//Ca). The cell was subjected to the same potentiostatic holds (−0.5 and −1.8 V) that were used for analyzing the plating potentials of the calcium symmetric cell. Following the potentiostatic holds, the gold electrodes were analyzed with XRD and SEM/EDX. Figure 4a,b shows the ex-situ XRD study performed on the gold working electrodes. The established reflections for gold are 38°, 44.4°, 64.7°, and 77.8°. All other reflections are from the deposition of materials onto the surface from plating with KPF_6_ electrolyte. Three phases were observed from the plating potential at −0.5 V including Ca, P_2_O_5_, and residual KPF_6_. The calcium reflection is observed at 28.27°. The P_2_O_5_ phase reflections are at 17.27° and 35°. The residual KPF_6_ on the gold electrode surface is indicated by the reflections at 19.8°, 20°, and 20.2°. The plating at −1.8 V produced the same potassium and potassium difluorodiphosphate phases that were observed with the calcium symmetric cell. A potassium phase reflection is observed at 28.8° while the K_2_P_2_O_5_F_2_ phase is outlined by reflections at 19.6°, 20.9°, 26.1°, 30.3°, and 31.4°. To verify that all activity from the potentiostatic holds were strictly plating behavior, a linear stability window experiment was performed (Figure S3). The KPF_6_ electrolyte remained stable with the gold electrode up to 4 V, confirming that no side reactions are occurring on the gold electrode.

Figure 4c is the SEM/EDX analysis performed on the gold working electrodes postplating. The calcium, oxygen, fluorine, phosphorus, and potassium atomic signals were recorded to be 2.2%, 15.59%, 15.45%, 1.09%, and 3.2%, respectively. The phosphorus, potassium, and fluorine signals would account for the residual KPF_6_ on the electrode. The remaining calcium signal would be due to the plating of calcium metal onto the gold electrode. The morphology of the deposited materials at −0.5 V is characterized as small globules. When analyzed with an EDX point spectra, the globule was found to have a calcium atomic signal of 42%, confirming that the composition of the plated calcium is calcium metal. When the plating potential of the KPF_6_ system was increased to −1.8 V, the SEM/EDX analysis revealed a potassium atomic signal increase from 3.2% at −0.5 V to 15.97% at −1.8 V. The signals for calcium, oxygen, fluorine, and phosphorus were reported to be 0.41%, 38.59%, 8.3%, and 1.38%, respectively. Additionally, the deposition of potassium on the gold working electrode surface also had more of a fractal pattern to it. When comparing the current densities from the two plating potentials, the −0.5 V plating achieved a steady current density of 0.00015 mA/cm^2^ while the −1.8 V plating had a current density of 0.112 mA/cm^2^. The differences between current densities are orders of magnitude apart from one another and consequently produce plated materials with drastically different morphologies.^31^

**Figure 5:**
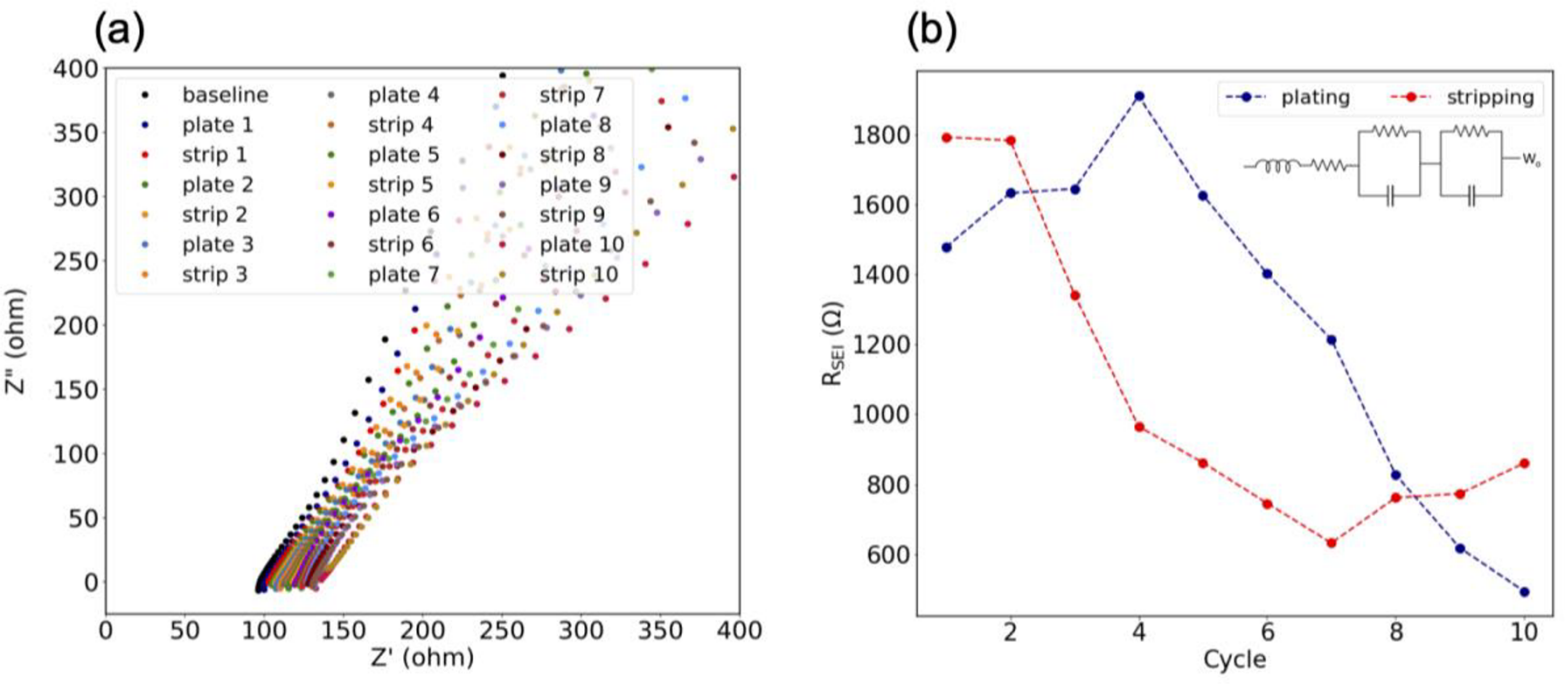
In-situ EIS of calcium electrodes during galvanostatic cycling. (a) Nyquist plot from in-situ EIS. (b) Hybrid SEI resistance from in-situ EIS. Inset in (b) is equivalent circuit for SEI resistance.

To maintain such low overpotentials throughout the course of plating and stripping, the SEI itself should have low resistance. The resistance of the SEI was studied through the use of in-situ EIS where the impedance of the SEI was studied as a function of cycling. The first 10 cycles of plating and stripping were studied on the calcium electrode surfaces, and the resulting Nyquist plot is shown in Figure 5a. The full scale of the Nyquist plot can be found in Figure S4. An equivalent circuit was used to determine the SEI resistance, the schematic of which is the inset of Figure 5b. The circuit includes an inductor which accounts for impedance at high frequencies. The first resistor in the circuit accounts for the bulk resistance of the electrode. Following the first resistor, the first parallel circuit describes the interfacial resistance and the second parallel circuit models the charge transfer. A Warburg element was also included in the equivalent circuit.^32−36^Figure 5b summarizes the SEI resistance over the course of cycling with the KPF_6_ electrolyte between the calcium symmetric cell. The SEI resistance increased to above 1800 Ω on the first cycles of testing, consistent with the formation of the SEI on the calcium electrode surface. Following the formation of the SEI, the resistances decreased to 800 Ω by cycle 8. The plating resistance during testing was larger than the stripping resistance. This is consistent with the cycling behavior because the plating SEI would have a thicker layer and therefore a higher resistance. The decrease of the SEI resistance over subsequent cycles would indicate an increase in ionic conductivity.^5^ This increase in ionic conductivity is also observed as the evolution of the plating plateau at −0.2 V during the calcium symmetric cell plating and stripping experiment (Figure 1a). The overpotentials and capacities at the −0.2 V plateau increase over the lifetime of testing, consistent with the maximum current response observed in the cyclic voltammetry. The resistance of the SEI stabilized at the end of cycling, indicating the formation and retention of a stable SEI layer.

**Figure 6:**
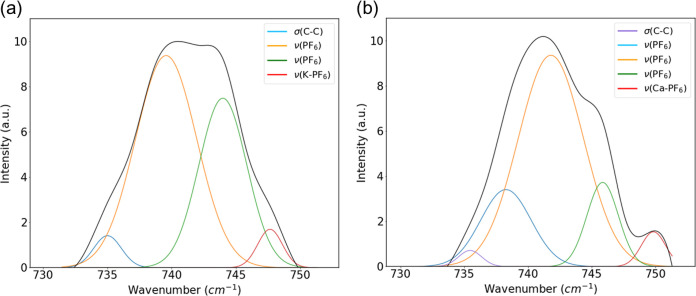
Raman analysis of electrolyte. (a) Gaussian fits of PF_6_^–^ interactions in KPF_6_ electrolyte (pristine). (b) Gaussian fits of PF_6_^–^ interactions in KPF_6_ electrolyte (cycled).

The last component analyzed was the KPF_6_ EC/DMC/EMC electrolyte. A transference number experiment was performed on a pristine electrolyte sample using protocols previously described.^17−20^ The transference number of the electrolyte was reported to be 0.101 (Figure S5). This value is consistent with other findings on the transference number of other potassium electrolytes.^37^ The low value of the transference number was indicative that the majority of ion movement is accomplished by the PF_6_^–^ anion. Focusing on the PF_6_^–^ anion interactions, Figure 6a shows the native electrolyte as analyzed under Raman spectroscopy. The results from peak deconvolution reveal a contact ion pair existing at 748 cm^–1^.^38^ This particular contact ion pair interaction was understood to be the interaction between K^+^ and PF_6_^–^ ions. Additionally, peaks are observed at 734, 739, and 744 cm^–1^. The peaks at 739 and 744 cm^–1^ are identified as symmetric PF_6_^–^ stretching and are consistent with the interactions of anions with the different solvents.^39,40^ The 734 cm^–1^ peak has been identified as the C–C bond in ethylene carbonate.^41^ Following 20 plating and stripping cycles in a symmetric cell, there is a shift of several peaks to higher wavenumbers. The peak at 735 cm^–1^ is the C–C bond in ethylene carbonate. Peaks observed at 738, 742, and 746 cm^–1^ are interactions of the PF_6_^–^ anion with the solvents of the electrolyte.^39,40,42^ The peak shift to 750 cm^–1^ is a contact ion pair that forms between the PF_6_^–^ anion and the uptake of calcium into the electrolyte.^43,44^ These shifts in wavenumbers outline how the continued plating and stripping with the hybrid SEI also produces compositional changes in the electrolyte, creating a dual ion system for plating and stripping. Previous research efforts mixing Li(BH_4_) and Ca(BH_4_)_2_ as a dual ion electrolyte promoted conversion of the Ca(BH_4_)_2_ into ionic clusters that were more electrochemically favorable for plating and stripping.^45^ Similar effects are observed with the evolution of capacities at 0.2 V over the course of cycling. While calcium plating does occur at both 0.2 and 1.8 V overpotentials, the redox activity benchmarked at 0.2 V was determined to be predominantly calcium plating and the 1.8 V overpotential was predominantly potassium. The increase of the capacity at 0.2 V overpotentials reflects the compositional change of the electrolyte and how it translates to increased redox activity at that potential. Inductively coupled plasma mass spectrometry (ICP-MS) was performed 70 h into plating and stripping with a symmetric cell. The results from the analysis revealed a calcium concentration of 0.004 M in the cycled electrolyte. As cycling of the cell would continue, increase of the calcium concentration in the electrolyte would be expected with the increased electrochemical activity at 0.2 V.

## Conclusion

We have shown successful plating and stripping with calcium metal electrodes using a native potassium electrolyte while maintaining overpotentials at 2 V. The SEI formed from the galvanostatic cycling plates and strips both calcium and potassium, as observed with XRD, FTIR, and EDX. The SEI formed on the calcium electrodes is influenced by the two regions of plating and stripping behavior with Ca, CaF_2_, and P_2_O_5_ phases forming at the 0.2 V overpotential and K_2_P_2_O_5_F_2_ and K phases forming at 1.8 V. The SEI also has a mixture of permanent and transient phases with K and K_2_P_2_O_5_F_2_ being removed after stripping while CaF_2_ and P_2_O_5_ remain. The uptake of calcium into the KPF_6_ electrolyte over the course of cycling transitions the conducting media to a dual ion system that increases the availability of calcium. The compositional changes to the electrolyte and low interfacial resistance of the SEI allow for increased plating and stripping of calcium and is most notable with the increased capacities at 0.2 V. This work provides demonstration that potassium electrolytes provide similar benefits to the plating and stripping of calcium that has been observed with sodium electrolytes. While the plating and stripping behavior of sodium electrolytes is more concise with all redox activity being observed at a single overpotential, the potassium electrolytes develop an SEI through a more multistep behavior. Future opportunities with this work can be explored with understanding the dynamics that lead to the development of two distinct regions of redox activity. Other features of this hybrid system that need to be addressed are strategies to lower the overpotentials from 1.8 V. Optimizing the electrolyte to function at 0.2 V is essential for more substantial calcium cycling and for the system to serve as an alternative to such electrolytes such as Ca(BH_4_)_2_ in THF. One such optimization that needs to be explored is different salt and solvent combinations. Another aspect of this hybrid SEI that needs further investigation is lifetime testing of the potassium electrolyte with higher current densities and capacities.

## Supporting Information Available

XRD of calcium metal, plating and stripping with three-electrode setup, linear stability window with gold electrode, EIS plots, and transference number measurement (PDF)
