# Supplementary material for: Plating and Stripping Calcium Metal in Potassium Hexafluorophosphate Electrolyte toward a Stable Hybrid Solid Electrolyte Interphase
Source: ACS Appl Energy Mater. 2023 Mar 30;6(7):3924–32. doi: 10.1021/acsaem.3c00098 (PMC10091900; doi:10.1021/acsaem.3c00098)
Supplement: Supplementary file 1 — ae3c00098_si_001.pdf [file ae3c00098_si_001.pdf]

SUPPLEMENTAL INFORMATION

Plating and Stripping Calcium Metal in Potassium Hexafluorophosphate Electrolyte Towards a Stable Hybrid Solid Electrolyte Interphase

Paul Alexis Chando,[1] Jacob Matthew Shellhamer, [1] Elizabeth Wall, [1] Wenlin He, [1] Ian Dean Hosein[1*]

1. Syracuse University, Department of Biomedical and Chemical Engineering, Syracuse, NY, 13244, United States

*Corresponding author: idhosein@syr.edu

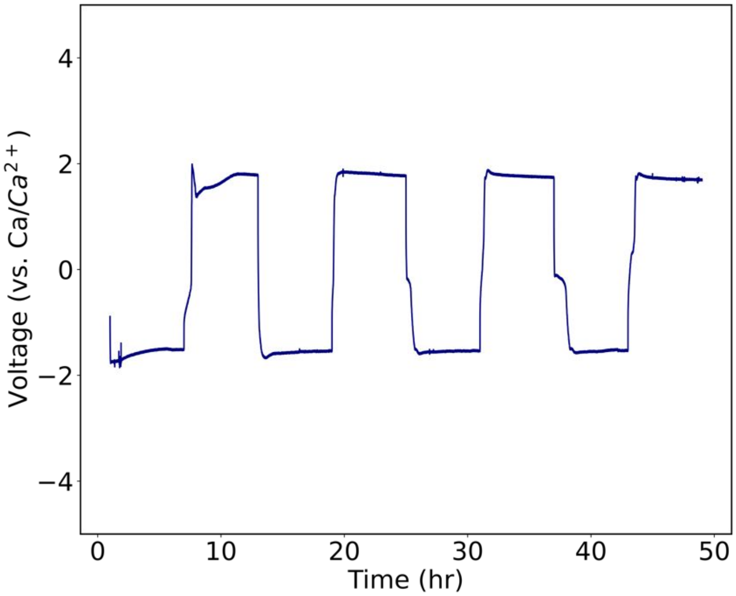

Figure S1 – Plating and stripping Ca//Ca symmetric cell with dedicated calcium reference electrode

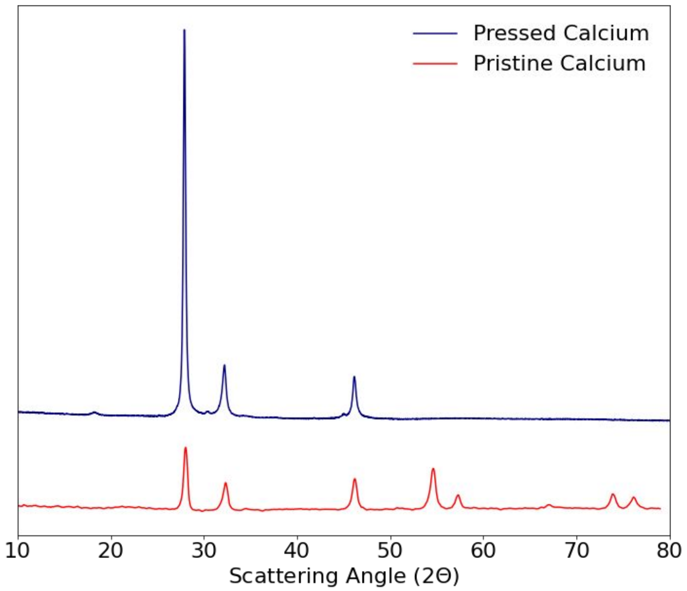

Figure S2 - XRD of pristine and pressed calcium pellets

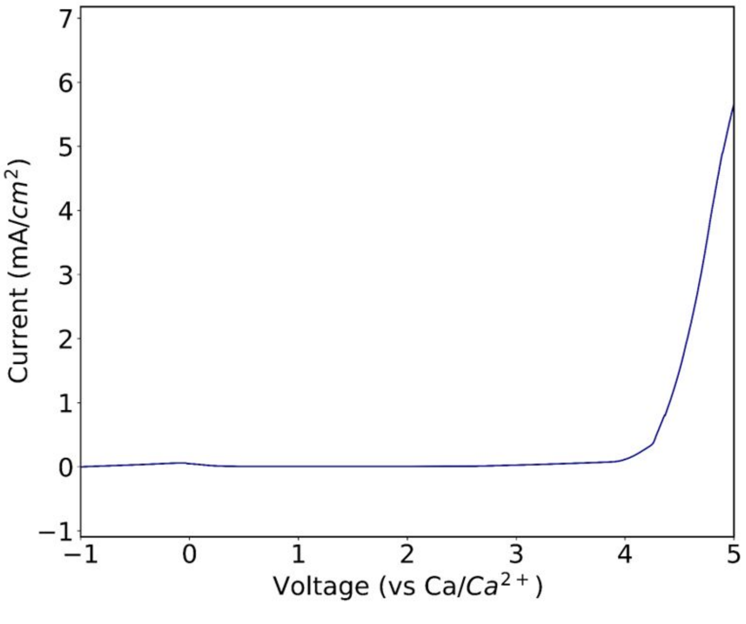

Figure S3 - Linear stability window of KPF_6 EC/DMC/EMC using a gold blocking electrode and calcium nonblocking electrode at 0.5 mV/s

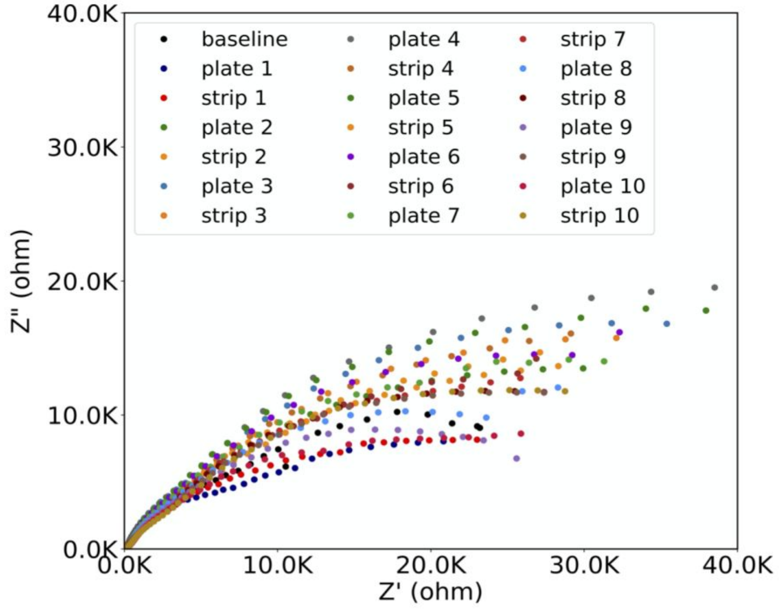

Figure S4 - Nyquist plots of impedance for galvanostatic cycling and after each plating and stripping

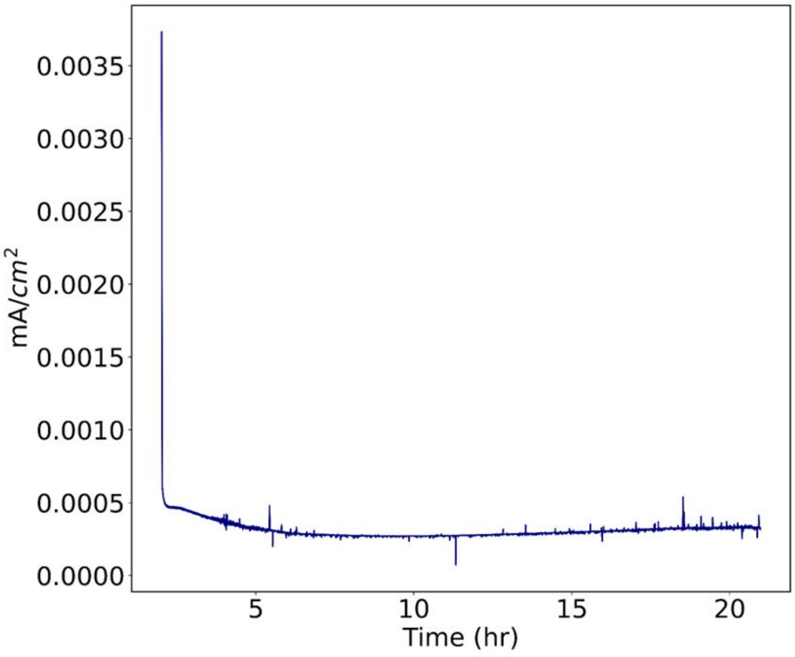

Figure S5 - Transference number measurement of KPF_6 EC/DMC/EMC (uncycled)
